# A Systematic Pan-Cancer Analysis of Genetic Heterogeneity Reveals Associations with Epigenetic Modifiers

**DOI:** 10.3390/cancers11030391

**Published:** 2019-03-20

**Authors:** Mafalda Ramos de Matos, Ioana Posa, Filipa Sofia Carvalho, Vanessa Alexandra Morais, Ana Rita Grosso, Sérgio Fernandes de Almeida

**Affiliations:** 1 Instituto de Medicina Molecular João Lobo Antunes, Faculdade de Medicina da Universidade de Lisboa, 1649-028 Lisboa, Portugal; mafaldarmatos@msn.com (M.R.d.M.); ioana.posa@gmail.com (I.P.); filipa.carvalho@medicina.ulisboa.pt (F.S.C.); vmorais@medicina.ulisboa.pt (V.A.M.); 2 UCIBIO, Departamento de Ciências da Vida, Faculdade de Ciências e Tecnologia, Universidade NOVA de Lisboa, 2829-516 Caparica, Portugal

**Keywords:** cancer, intratumor heterogeneity, genomic instability, epigenetics, mitochondrial metabolism

## Abstract

Intratumor genetic heterogeneity (ITH) is the main obstacle to effective cancer treatment and a major mechanism of drug resistance. It results from the continuous evolution of different clones of a tumor over time. However, the molecular features underlying the emergence of genetically-distinct subclonal cell populations remain elusive. Here, we conducted an exhaustive characterization of ITH across 2807 tumor samples from 16 cancer types. Integration of ITH scores and somatic variants detected in each tumor sample revealed that mutations in epigenetic modifier genes are associated with higher ITH levels. In particular, genes that regulate genome-wide histone and DNA methylation emerged as being determinant of high ITH. Indeed, the knockout of histone methyltransferase SETD2 or DNA methyltransferase DNMT3A using the CRISPR/Cas9 system on cancer cells led to significant expansion of genetically-distinct clones and culminated in highly heterogeneous cell populations. The ITH scores observed in knockout cells recapitulated the heterogeneity levels observed in patient tumor samples and correlated with a better mitochondrial bioenergetic performance under stress conditions. Our work provides new insights into tumor development, and discloses new drivers of ITH, which may be useful as either predictive biomarkers or therapeutic targets to improve cancer treatment.

## 1. Introduction

The expansion of genetically-distinct cell populations within a tumor creates a subclonal architecture that varies dynamically throughout cancer progression [1]. This acquired cancer trait, termed intratumor heterogeneity (ITH), is the substrate for Darwinian evolution to act upon, selecting subclones carrying phenotypes that favor tumor progression [2]. The outgrowth of such subclones impacts cancer development, drug resistance and tumor relapse [3,4,5,6]. Despite the key role ITH plays in cancer, important questions regarding its magnitude, origin and genetic drivers across different cancer types remain largely unanswered. By facilitating the emergence of nucleotide sequence mutations, copy-number alterations, chromosomal translocations or aneuploidies, genomic instability has been regarded as the major source of ITH [4,7,8,9]. However, discrepancies in the rates of genomic instability and ITH observed in previous comprehensive studies [3] suggest that additional events congregate to increase genetic heterogeneity in tumors.

Besides mutations, cancer cells invariably present with some degree of epigenetic alterations that contribute to the acquisition of the cancer hallmarks [10,11]. Indeed, there is evidence that epigenomic reprogramming plays a seminal role in tumorigenesis by creating a progenitor-like cell state that facilitates expression of driver mutations and tumor initiation [12]. High-resolution genome-sequencing efforts have identified driver mutations in genes that regulate the epigenome, namely, genome-wide chromatin and DNA methylation [13,14]. For instance, acute monocytic leukemias frequently (20.5%) carry mutations in the de novo DNA methyltransferase gene *DNMT3A*, displaying aberrant genome-wide DNA methylation profiles [15]. Ten percent of kidney renal clear cell carcinomas (KIRC) have mutations in *SETD2*, the methyltransferase responsible for trimethylation of Lys36 in histone H3 (H3K36me3), which is necessary for accurate gene expression and DNA repair [16,17,18,19]. H3K36me3 is also involved in targeting DNMT3A to chromatin [20], highlighting the finely tuned epigenetic interplay between histone and DNA methylation that is needed for normal cell function and is frequently disrupted in cancer cells.

While epigenetic deregulation in cancer arises primarily as a consequence of DNA mutations, the view that altered epigenomes may also change DNA mutation rates highlights reciprocal interactions that contribute to cancer development [14,21]. Accordingly, epigenomic disruption should favor the development of genetically-diverse tumor cell populations, fueling ITH [21]. In fact, a possible relationship between genomic and epigenomic alterations during clonal evolution of tumors has recently been suggested in esophageal squamous cell carcinoma and glioma, where high concordance was observed between the evolution of genetic and epigenetic diversification [22,23]. In this study, we reasoned that analysis of whole-exome datasets of The Cancer Genome Atlas (TCGA) would disclose patterns of covariation between specific epigenetic modifier genes and ITH levels. Our integrative pan-cancer characterization of somatic variants and ITH identified mutations in epigenetic modifier genes that display an association with increased clonal evolution across several cancer types. Experimental ablation of specific *loci* provided direct evidence that loss of *SETD2* or *DNMT3A* drives the emergence of genetically-distinct subclonal cell populations. Knockout cells showed increased mitochondrial bioenergetic performance under stress conditions, a phenotypic trait that fosters the Darwinian selection of clones. Our results provide an unprecedented pan-cancer portrait of the major determinants of ITH and an experimental validation of the role of specific epigenetic modifier genes, laying a foundation for more effective cancer prognoses and treatment.

## 2. Results

### 2.1. Genomic Instability Does Not Predict ITH in Many Cancer Types

**Figure 1:**
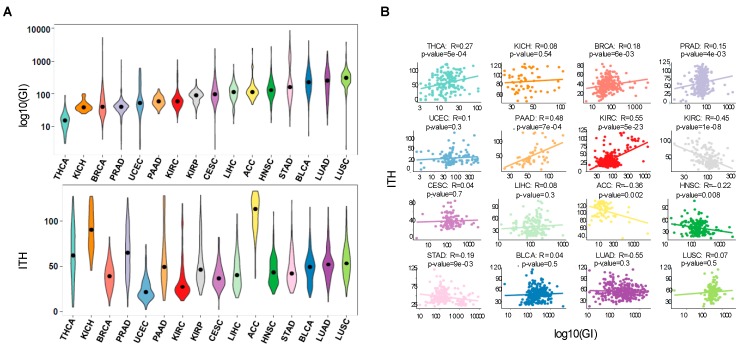
Pan-cancer correlations reveal that genomic instability does not predict ITH. (**A**) Distribution of genomic instability (log10 transformed) and ITH across 16 TCGA cancer types: THCA (thyroid carcinoma), KICH (kidney Chromophobe), BRCA (breast invasive carcinoma), PRAD (prostate adenocarcinoma), UCEC (uterine Corpus Endometrial Carcinoma), PAAD (pancreatic adenocarcinoma), KIRC (kidney renal clear cell carcinoma), KIRP (kidney renal papillary cell carcinoma), CESC (cervical squamous cell carcinoma and endocervical adenocarcinoma), LIHC (liver hepatocellular carcinoma), ACC (adrenocortical carcinoma), HNSC (head and neck squamous cell carcinoma), STAD (stomach adenocarcinoma), BLCA (bladder urothelial carcinoma), LUAD (lung adenocarcinoma), LUSC (lung squamous cell carcinoma). Cancers are ordered according to genomic instability levels. (**B**) Pearson correlation between genomic instability (log10 transformed) and ITH for each cancer type. Each point represents one patient and the line shows the fitted linear model.

To estimate correlations between genomic instability and ITH in different cancers, we examined 2807 tumor whole-exome sequences from 16 cancer types of TCGA. We assigned an overall genomic instability score to each tumor, defined as the number of somatic point mutations and small insertions and deletions (INDELs) ranging from 1 to 100 bp in length. The ITH score was obtained using the mutant-allele tumor heterogeneity (MATH) method (Figure 1A and Table S1) [24]. MATH evaluates the variability of the mutant-allele fractions among all tumor-specific mutated *loci*. Therefore, homogeneous tumors with high mutation incidence have a narrower distribution of mutant-allele fractions than heterogeneous tumors. In agreement with previous reports [3], we found that the degree of genomic instability is highly variable across tumors types (Figure 1A). Notably, high levels of genomic instability were not positively correlated with ITH in several tumors (Figure 1B). Individual analysis of each cancer type revealed that only thyroid carcinoma (THCA), pancreatic adenocarcinoma (PAAD) and kidney renal clear cell carcinoma (KIRC) exhibited a statistically significant positive correlation between genomic instability and ITH (Figure 1B). Moreover, we found a significant negative correlation between these two features in kidney renal papillary cell carcinoma (KIRP) and adrenocortical carcinoma (ACC) (Figure 1B). This finding suggests that factors other than increased mutability determine the development and expansion of genetically-distinct subclonal cell populations within a tumor.

### 2.2. Mutations in Epigenetic Modifier Genes Are Strong Determinants of ITH

**Figure 2:**
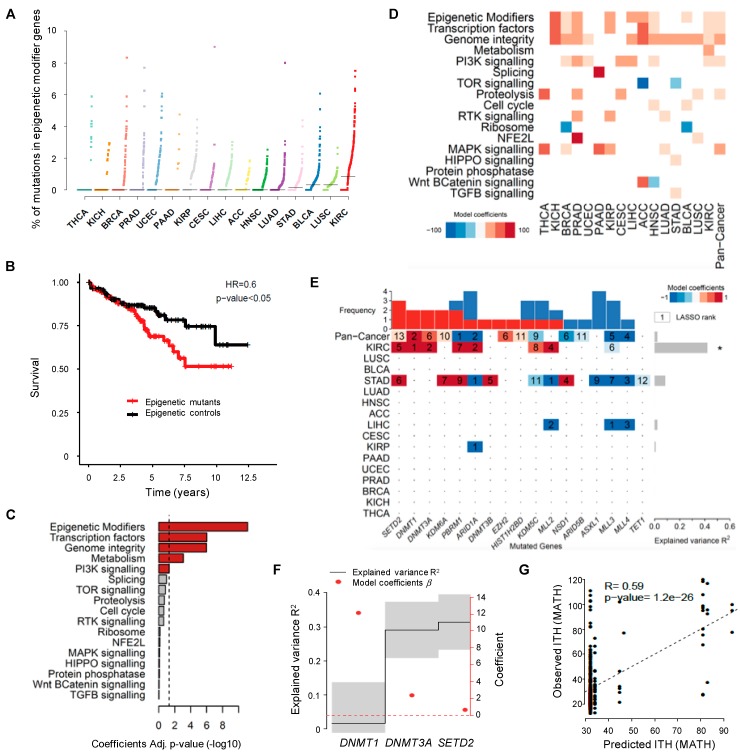
Driver mutations of pan-cancer ITH. (**A**) Pan-cancer analysis of the percentage of somatic mutations in epigenetic modifier genes across 16 TCGA cancer types. The vertical axis shows the percentage of mutations in epigenetic modifier genes whereas the different cancer types are ordered on the horizontal axis from the lowest to the highest percentage of mutations in these genes. (**B**) Kaplan-Meier plot comparing the survival of KIRC patients segregated according to the presence (red) or absence (black) of mutations in epigenetic modifiers. The log-rank test was used for statistical analysis. (**C**) Statistical significance (−log 10 Benjamini-Hochberg Adj. *p*-value) of the linear model coefficients estimated for each gene group in KIRC. The vertical dashed line corresponds to the significance level (BH adj. *p*-value of 0.05). (**D**) Heatmap of the linear model coefficients estimated for each cancer type and gene group. Only statistically significant coefficients are represented (BH adj. *p*-value < 0.05). (**E**) Heatmap of driver mutations of ITH across several cancer types depicted by a LASSO penalized model. LASSO-selected coefficients are colored according to the effect of each standardized covariate in the optimal model. The numbers on each tile denote the order in which variables are included indicating their relative importance. The top bar plot indicates the frequency at which each driver-gene mutation occurs in the ITH fitted model. The right bar plot shows the explained variance. An asterisk (*) denotes models where the explained variance (R^2^) is greater than zero by a margin of more than one standard deviation. (**F**) Variance explained by selected driver genes (black line ± 1 standard deviation) ordered by their occurrence in a LASSO penalized model for ITH in KIRC using only the mutated genes *DNMT1*, *DNMT3A* and *SETD2*. The optimal model maximizes the explained variance R2. The right axis indicates the effect of each standardized covariate in the optimal model (red dots). (**G**) Scatter plot of predicted and observed ITH for KIRC (Estimate and statistical significance of the Pearson correlation are presented).

To investigate whether epigenomic deregulation drives the development of tumors with high levels of ITH, we focused our analysis on KIRC, the cancer type with the highest frequency of mutations in epigenetic modifiers (Figure 2A). The important role of epigenomic deregulation in the development and progression of KIRC is illustrated by the finding that patients with mutations in epigenetic modifiers have worse overall survival compared to those without mutations in these genes (*p* < 0.05, log-rank test; Figure 2B). To investigate how epigenomic deregulation compares with other specific cellular processes in influencing ITH in KIRC, we analyzed significantly mutated genes grouped in broad functional categories as previously described [25]. The linear model revealed that mutations in epigenetic modifiers are the most strongly associated with high ITH in KIRC, amongst all categories of genes analyzed (Figure 2C). Moreover, the presence of mutations in epigenetic modifier genes correlates positively with increased ITH across different cancer types (Figure 2D and Table S2). Next, we aimed at identifying the individual genes that, when mutated, more accurately predict ITH. To this end, we used generalized linear models previously applied to infer the association of genetic alterations with other phenotypic variables [26]. The strongest predictor of high ITH in both KIRC alone or across several cancer types was the presence of mutations in *SETD2*, *DNMT1* and *DNTM3A* (Figure 2E). Importantly, we could model 32% of variability in KIRC ITH using only mutations in *SETD2*, *DNMT1* and *DNTM3A* (Figure 2F). The optimal model showed a significant correlation between the observed and predicted ITH levels based on the tumor mutation profiles (Figure 2F,G). These data suggest that epigenomic deregulation is an important determinant of ITH and identify mutations in *SETD2*, *DNMT1* and *DNTM3A* as candidate drivers of ITH.

### 2.3. Knockout of SETD2 or DNMT3A Expands the Clonal Diversity of Cancer Cell Populations

**Figure 3:**
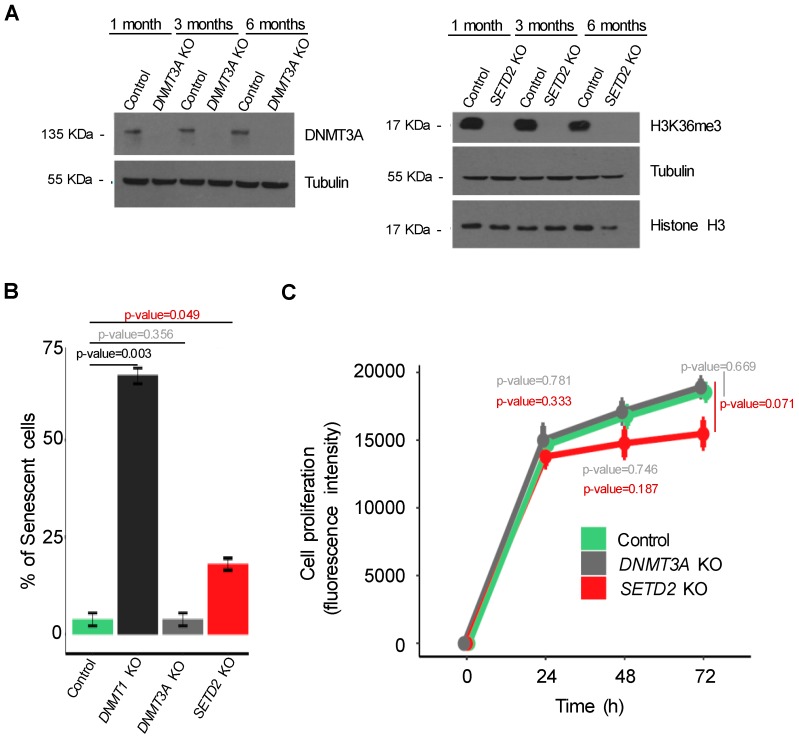
CRISPR/Cas9 knockout of candidate ITH-driver genes in cancer cells. (**A**) The levels of DNMT3A and H3K36me3 were estimated by western blot 1, 3 and 6 months after knockout. (**B**) The percentage of senescent cells in control and mutant conditions (*SETD2, DNMT1* and *DNMT3A* knockouts) was assessed by β-galactosidase staining (error bars indicate SEM; *n* = 3 counting regions of 150 cells/condition in triplicate; Student *t*-test). (**C**) The proliferation rate of the indicated cells was measured by AlamarBlue dye reduction at the indicated time points. All data are presented as mean (four technical replicates in the same experiment) ± SEM.

We next sought to experimentally validate the role of *SETD2*, *DNMT1* and *DNMT3A* mutations in driving the emergence of genetically-distinct subclonal cell populations. The mutations found in these genes were predicted as deleterious causing loss of function (Table S3). To recapitulate this phenotype, we employed CRISPR/Cas9 system to specifically knockout each of these genes in KIRC Caki-2 cell lines. Insertion of small INDELs at the target sites was confirmed by DNA sequencing and efficiency of gene knockout evaluated by measuring protein levels (Figure 3A). Decreased H3K36me3 levels were used as a surrogate for SETD2 depletion (Figure 3A). Importantly, knockout of DNMT1 rendered KIRC cells senescent (Figure 3B), in contrast to DNMT3A and SETD2 depletion, which were well tolerated and did not significantly affect cell proliferation (Figure 3C). This finding suggests that additional compensatory mutations are required to allow the proliferation of *DNMT1* mutant cells within tumors. Alternatively, *DNMT1* mutant clones could be selected during tumor evolution due their ability to promote carcinogenesis through the senescence-associated secretory phenotype [27,28,29].

**Figure 4:**
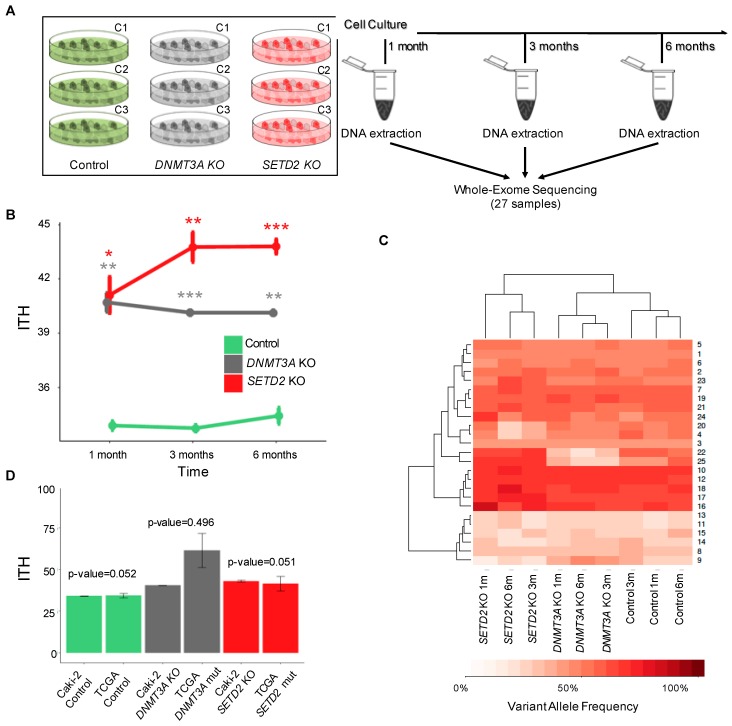
*SETD2* and *DNMT3A* knockout drive ITH. (**A**) Schematic representation of the experimental setup. Control and knockout cells were cultured during the indicated time periods before DNA extraction and whole-exome sequencing (WES). ITH was inspected after three independent clonal expansions (C1–C3) for each knockout at each time point. (**B**) ITH levels of *SETD2* and *DNMT3A* knockout cells after 1, 3 and 6 months. WES data of the indicated conditions were used to calculate ITH, as described in the Methods. Data from three independent clonal expansions analyzed per group are presented as mean ± SEM. Statistical analysis was a two-tailed Student’s *t*-test (* *p* < 0.05, ** *p* < 0.01, *** *p* < 0.001). (**C**) Hierarchical cluster analysis of the mean variant allele frequency estimated with PyClone in control, *SETD2* and *DNMT3A* knockout cells. (**D**) Distribution and comparison of the ITH levels across KIRC patients from TCGA and Caki-2 cell lines for the indicated conditions (control, *SETD2* and *DNMT3A* knockouts). The bar graph displays mean ITH values and s.e.m. (standard error of the mean). Statistical analysis was performed with Wilcox-test but no statistical significance was observed between TCGA patients and each cell line.

To investigate whether loss of *DNMT3A* or *SETD2* drives the acquisition of genetically-heterogeneous cell populations over time, we performed whole-exome sequencing of control and knockout cells cultured during 1, 3 and 6 months (Figure 4A). ITH levels of three different cell populations per experimental condition (control, *SETD2* and *DNMT3A* knockout) were measured at each time point using MATH. Compared to control cells, loss of either *SETD2* or *DNMT3A* resulted in significantly increased and comparable levels of ITH after just one month (Figure 4B and Table S4). However, while ITH rose for up to three months after *SETD2* depletion, it remained constant through time in *DNMT3A* knockout cells (Figure 4B). Bayesian cluster analysis of mutations using PyClone [30] identified 25 mutation clusters that are distributed in each cell population at a frequency that permits segregation according to the knockout gene (Figure 4C). ITH scores observed in *SETD2* and *DNMT3A* knockout cell lines were not significantly different from those determined in TCGA samples carrying *SETD2* and *DNMT3A* mutations, respectively (Figure 4D). This finding reveals that the clonal dynamics of cancer cells grown in vitro recapitulates the in vivo scenario. Altogether, these data suggest that loss of *SETD2* or *DNMT3A* drives specific patterns of clonal evolution that culminate in tumors with increased levels of ITH.

### 2.4. Epigenomic Deregulation Drives Favorable Metabolic Phenotypic Variation

**Figure 5:**
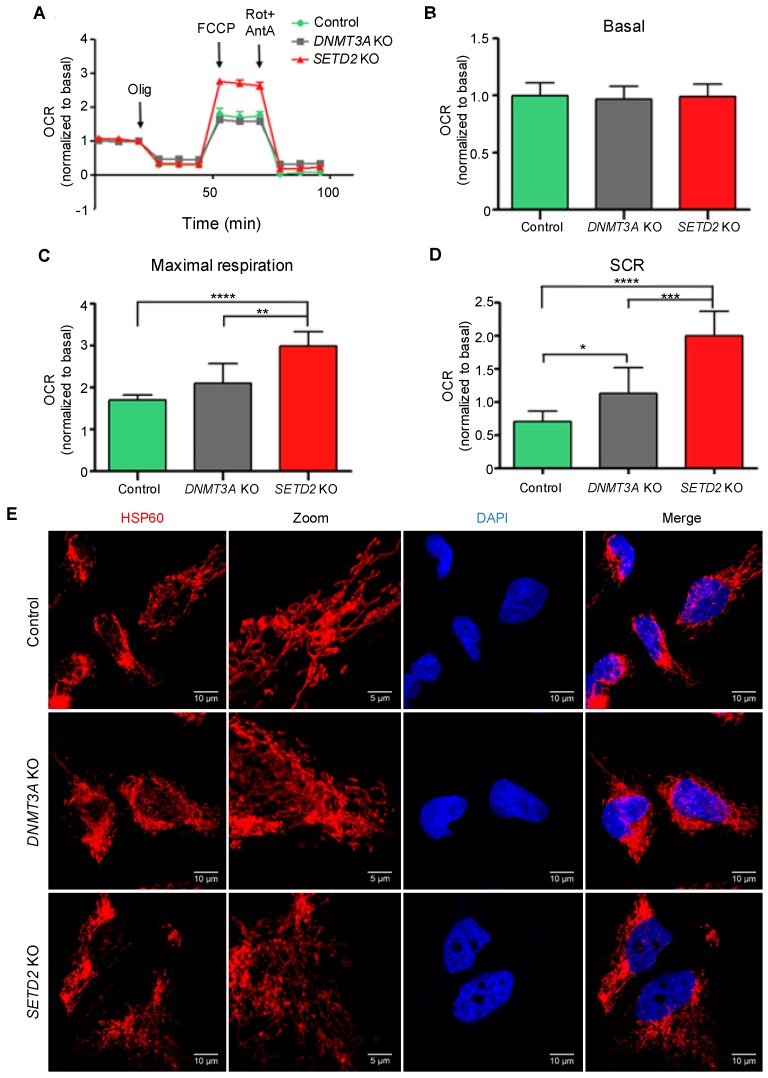
*SETD2* and *DNMT3A* knockout increase bioenergetic performance. Oxygen Consumption Rates (OCR) trace and respiration parameters were measured in control, *SETD2* and *DNMT3A* knockout cells. Seahorse extracellular flux measurements of OCR was normalized to basal respiration (**A**). Basal respiration (**B**), maximal respiration (**C**) and spare capacity rate (SCR) (**D**) of Caki-2 cell lines were obtain by OCR values representative of 3 independent experiments in which each data point represents replicates of three to five wells each cell line. Statistical analysis was performed using the unpaired Student’s *t*-test, where * *p* < 0.05; ** *p* <0.01; *** *p* < 0.001; **** *p* < 0.0001, data were represented as the mean ± SD. Olig: Oligomycin; FCCP; carbonyl cyanide-4-(trifluoromethoxy)phenylhydrazone; Rot+AntA; Rotenone+Antimycin A. (**E**) Mitochondria morphology of Caki-2 control, *DNMT3A* KO and *SETD2* KO cell lines. Cells were fixed and stained with the mitochondrial marker Hsp60 (red) and with the nucleus marker DAPI (blue). Cells were imaged on an inverted Zeiss LSM 880 microscope. Fiji software was used to calculate scale bar (10 µm or 5 µm for zoom-in). Selected image is representative of three independent experiments.

The increased ITH observed knockout of *SETD2* or *DNMT3A* knockout suggests that new clones carrying phenotypic traits that confer selective advantage within the cell populations have expanded and were selected. In cancer cells, mitochondria play important roles in energy production, redox and calcium homeostasis, transcriptional regulation and cell death [31]. Changes in mitochondrial metabolism constitute an important source of variability for natural selection to act upon [32,33]. To test whether epigenomic deregulation drives altered mitochondrial metabolic functions, we evaluated the ability of cells to adapt to shifts in energy demands by measuring mitochondrial respiration rates using an oxygen electrode on the Seahorse platform. In this assay, the oxygen consumption rate was measured before and after the addition of inhibitors to derive parameters of mitochondrial respiration in baseline and stress conditions (Figure 5A). Basal mitochondrial respiration in knockout and parental cells was equally efficient (Figure 5B), indicating that no major intrinsic metabolic alterations were caused upon loss of either *SETD2* or *DNMT3A*. We then measured the maximal respiratory capacity and spare capacity rate (SCR) of cells challenged with the mitochondrial uncoupler FCCP and the Complex I and Complex III specific inhibitors rotenone and antimycin A, respectively. Both parameters were significantly increased in *SETD2* and *DNMT3A* knockout cells when compared to parental cells under similar conditions (Figure 5C,D). Analysis of *SETD2* and *DNMT3A* knockout cells revealed mutations in genes involved in mitochondria biogenesis and function (Table S5); however, inspection of mitochondria network in knockout cells using fluorescence confocal microscopy did not reveal any major alterations (Figure 5E). These data rule out altered morphology as a causing factor for the observed increase in the spare capacity rate. Instead, our data suggest that gain-of-function mutations in genes involved in mitochondrial function drive higher spare capacity rates in knockout cells. Such an association between epigenetics, altered nuclear DNA expression and mitochondrial function has already been demonstrated in previous studies [34]. Altogether, these data provide direct experimental evidence for the emergence of favorable characteristics in *SETD2* and *DNMT3A* depleted cells that may foster the increased number of genetically-distinct clones within the cell population.

## 3. Discussion

Tumors evolve through multiple rounds of clonal expansion, diversification and selection that enable the acquisition of metabolic and bioenergetic phenotypes better adapted to the local microenvironment. Such evolutionary adaptation also accounts for therapeutic failure as drug-resistant tumor clones may be selected during therapy. High ITH is the substrate for this Darwinian model of cancer evolution and therapeutic resistance, and hence, highlights the need for further understanding of drivers and mechanisms of clonal evolution. Despite the major discrepancies observed in their covariance rates [3], genomic instability is still considered a major source of ITH [4,7,8,9]. In this study we show that genomic instability is not positively correlated with ITH in most cancer types. In fact, there is a significant negative correlation in some cancers, suggesting that additional processes must congregate to drive genetic heterogeneity. Our results are in agreement with previous studies, where ITH was associated with different forms of instability [35]. Recently, high concordance was observed between the evolution of genetic and epigenetic diversification in esophageal squamous cell carcinoma and in glioma, disclosing possible relationships between genomic and epigenomic alterations during the clonal evolution of tumors [22,23]. An interesting hypothesis linking DNA mutations and epigenetics in cancer is that altered DNA methylation or chromatin modifications may accelerate mutation rates. Examples of such relationship were already described. For example, abnormal DNA hypomethylation near guanine quadruplexes (G4s)-rich regions is a common signature for many DNA breakpoints associated with somatic copy-number alterations [36]. This finding suggests that DNA hypomethylation in genomic regions enriched for G4s acts as a mutagenic factor in cancer. Additionally, the genome organization into heterochromatin and euchromatin-like domains is a dominant determinant of mutation rates, as illustrated by the finding that H3K9me3 levels alone can predict over 40% of somatic mutation *loci* in human cancer samples [37]. Conversely, we and others have shown that H3K36me3 protects active coding sequences of the genome from error-prone DNA double-strand break repair mechanisms by promoting homologous recombination [17,38,39]. Together, these data establish a strong association between epigenomic deregulation—namely, DNA and histone methylation and genomic mutations, which we show play important roles during clonal evolution and genetic diversification of tumors. In fact, we found that mutations in epigenetic modifier genes are the strongest determinants of ITH amongst a panel of 17 distinct cellular pathways. Particularly, we identified and validated mutations in the methyltransferase genes *SETD2* and *DNMT3A* as potent drivers of ITH. Other epigenetic modifiers were also associated with high levels of ITH in KIRC (e.g., *PBRM1* or *KDM5C*), but correlated with lower heterogeneity in a pan-cancer analysis or in other cancer types. Our findings add direct experimental evidence to previous studies implicating SETD2 loss-of-function in mechanisms that generate ITH [40,41].

As tumor cells adapt to the environment, they acquire distinctive bioenergetic features to take advantage of available fuels. For instance, tumor cells growing in an environment rich in adipocytes could use fatty acids as a major energy source [33]. This remarkable versatility arises from clonal evolution, during which genetic heterogeneity would eventually impact the function of metabolic enzymes [32,33]. We thus reasoned that the increased ITH observed upon *SETD2* or *DNMT3A* knockout likely underpins phenotypic variations in mitochondrial metabolism upon which natural selection could act. In agreement with this, we observed that both *SETD2* and *DNMT3A* depleted cell populations have increased bioenergetic performance under stress conditions, a phenotype that was accompanied by mutations in genes involved in mitochondria function.

## 4. Materials and Methods

### 4.1. Cell Culture

Caki-2 cells (Cell Line Services, Eppelheim, Germany) that do not have *SETD2* mutations were selected as a cellular model of KIRC. Caki-2 and human embryonic kidney (HEK) 293T (ATCC, Manassas, VA, USA) cells were grown as monolayers in Dulbecco’s modified Eagle medium (DMEM, Invitrogen, Carlsbad, CA, USA), supplemented with 10% (*v/v*) FBS, 1% (*v/v*) nonessential amino acids, 1% (*v/v*) L-glutamine and 100U/mL penicillin-streptomycin and maintained at 37 °C in a humidified atmosphere with 5% CO_2_.

### 4.2. Gene Knockout by CRISPR/Cas9

To establish knockout cell lines, we used the genome editing one vector system (lentiCRISPR-v2) (Addgene #52961). sgRNAs were designed by GenScript and the potential off-target effects was confirmed using the CRISPR tool (http://crispr.mit.edu). The following sgRNA sequences were selected: *DNMT1* CRISPR guide RNA 1: CTAGACGTCCATTCAC TTCC; *DNMT3A* CRISPR guide RNA 2: TGGCGCTCCTCCTTGCCACG and *SETD2* CRISPR guide RNA 1: AGTTCTTCTCGGTGTCCAAA. As a control we used a pCas-Scramble CRISPR Vector (SantaCruz, sc-418922). Recombinant lentiviruses were produced by co-transfecting HEK293T cells with each lentiCRISPR-v2 expression plasmid together with packaging plasmid pCMV-dR8.91 (Addgene) and the envelope plasmid pCMV-VSV-G (Addgene #8454) using Lipofectamine™ 3000 (Thermo Fisher Scientific, Waltham, MA, USA) as a transfection reagent and Opti-MEM (Invitrogen), according to the manufacturer’s instructions. Infectious lentiviruses were collected 48 h after transfection. The supernatant was filtered through 0.45 μm filters (GE Healthcare, Chicago, IL, USA) and concentrated by ultra-centrifugation at 25,000 rpm, 4 °C for 90 min. Cells were infected with lentivirus at approximately 60% confluence. After 24 h, cells were incubated with 5 µg/mL of puromycin (InvivoGen, San Diego, CA, USA) for 2 days. To identify KO clones, infected cells were single-cell cloned in 96-well plates. Several clones from 96-well plates were selected and the presence of DNMT1, DNMT3A and SETD2 was verified by western blot and Sanger sequencing. Genomic DNA was extracted from each clone and a segment surrounding the *DNMT1*, *DNMT3A* and *SETD2* edited region was amplified with specific primers (Table S6). Target sites and specificity were validated using the UCSC Genome Browser (https://genome.ucsc.edu/).

### 4.3. Western Blot

Whole cell protein extracts were prepared by cell lysis with SDS-PAGE buffer (80 mM Tris-HCL pH 6.8, 16% glycerol, 4.5% SDS, 450 mM DTT, 0.01% bromophenol blue) with 200 U/mL benzonase (Sigma-Aldrich, St. Louis, MO, USA), 50 μM MgCl2 and were boiled for 5 min. Equal amounts of protein extracts were resolved by SDS-polyacrylamide gel electrophoresis (SDS-PAGE) and transferred to a nitrocellulose membrane. After 1 h blocking with 5% non-fat dry milk in 1× PBS, 0.1% Tween20 at room temperature, membranes were incubated with antibodies as follows: anti-DNMT1 (2 µg/mL, Active Motif, Carlsbad, CA, USA), anti-DNMT3A (1:1000, Cell Signaling), anti-H3K36me3 (1:500, Abcam, Cambridge, UK), α-tubulin (1:15,000, Sigma-Aldrich) and anti-histone H3 (1:1000, Abcam). Detection was performed with the appropriate secondary antibodies (Bio-Rad, Hercules, CA, USA) and enhanced luminescence substrate (Pierce ECL, Thermo Fisher Scientific, Waltham, MA, USA). Details of antibodies used are mentioned in Table S6.

### 4.4. Cell Senescence and Proliferation Assays

Senescent cells were identified by β-galactosidase staining in low-density culture. Caki-2 cells (controls and KOs) were seeded in 6-well plates at 10 × 10^4^ cells/cm^2^. In the next day, cells were washed with PBS 1×, fixed for 5 min (RT) in 2% formaldehyde/0.2% glutaraldehyde, washed, and incubated at 37 °C (with no CO_2_) with senescence cells histochemical staining kit (Sigma-Aldrich, CS0030) according to manufacturer’s recommendations for 12 h. Blue-stained cells and total number of cells was counted under the phase contrast microscope (Leica DM2500, Leica Biosystems, Wetzlar, Germany).

Cellular proliferation for human cancer cell lines (controls and KOs) was measured every 24 h for four days, using AlamarBlue™ (Thermo Fisher Scientific). Briefly, 10 × 10^4^ cells/well were seeded on 96-well plates in a final volume of 100 μL per well. This is a reliable method for measuring cell viability, using the metabolic activity of cells to reduce resazurin (oxidized form: 7-hydroxy-3H-phenoxazin-3-1-10-oxide) to resorufin. The fluorescence of these two forms is measured at 560 nm as excitation wavelength and at 590 nm emission wavelength was measured every 24 h for 72 h, using a microplate reader (Microplate Reader TECAN Infinite M200, Tecan, Mannedorf, Switserland).

### 4.5. Mitochondria Oxygen Consumption Rate

Mitochondria oxygen consumption rate (OCR) was measured with the XF24 Extracellular Flux Analyzer (Seahorse Bioscience, Agilent, Santa Clara, CA, USA), according to the standard protocol. Briefly, at least 3 months after each knockout, cells were seeded one day prior to the assay in a 24-well XF plates at a density of 2 × 10^5^ cell/well and incubated overnight at 37 °C, 5% CO_2_. Twenty-four hours later, cells were incubated with Seahorse XF Base medium supplemented with 10 mM glucose, 2 mM L-glutamine and 1mM sodium pyruvate at pH 7.4 and calibrated for 1 h at 37 °C in the absence of CO_2_. Hydration of the sensor cartridge was performed one day prior to the assay at 37 °C in the absence of CO_2_. OCR was evaluated in a time course set-up where the following compounds were sequentially injected in the following order: oligomycin (1 µM final concentration), carbonyl cyanide-4-(trifluoromethoxy)phenylhydrazone (FCCP) (0.5 µM final concentration), and rotenone plus antimycin A (0.5 µM final concentration). Rates were normalized to protein concentration measured according to the Bradford method (Bio-Rad, Hercules, CA, USA). Three to five wells from each cell line were measure in a total of *n* = 3 experimental assays. Values for each parameter were calculated as the difference of OCR measures after and before injection:

- Non-mitochondrial respiration was calculated as the average of OCR measurements after rotenone and antimycin A injection;
- Basal respiration is calculated as the difference between non-mitochondrial respiration and the third point of baseline cellular oxygen consumption;
- Maximal respiration corresponds to the difference between the average OCR value after FCCP injection and the non-mitochondria respiration;
- Spare capacity rate (SCR) is the difference between maximal and basal respiration values.

### 4.6. Determination of Mitochondrial Morphology

Caki-2 control, Caki-2 DNMT3A and Caki-2 SETD2 cells were seeded on 13 mm coverslips. Twenty-four hours post seeding, cells were washed three times in PBS, fixed in 4% paraformaldehyde for 20 min, washed three times in PBS, permeabilized in 0.1% Triton X-100 in PBS for 10 min, followed by three washes in PBS. Cells were blocked in blocking buffer (0.2% gelatin, 2% fetal bovine serum, 2% BSA, 0.3% bovine serum albumin, 0.3% Triton X-100 in PBS) with 5% goat serum (DAKO) for 1 h. Cells were stained using the primary antibody mouse anti-hsp60 at 1/250 dilution (BD Bioscience) for 2 h. After 3 washes in PBS, cells were incubated with the secondary antibody Alexa Fluor 568 goat anti-mouse at 1/500 dilution (Life Technologies, Carlsbad, CA, USA) for 1 h and with DAPI at 1/10,000 dilution for 10 min. Images were visualized with a confocal laser point-scanning microscope Zeiss LSM 880 with airyscan through an objective of 63× 1.40 oil dipping lens (Zeiss, Oberkochen, Germany). Images were acquired using the ZEN software package (Zeiss) and analyzed in open source Fiji software (https://fiji.sc/).

### 4.7. Pan-Cancer Data Sets

WES data published in the context of TCGA was downloaded from Broad Institute MAF dashboard https://confluence.broadinstitute.org/display/GDAC/MAF+Dashboard, released (14 April 2017). A total of 2807 patients corresponding to 16 different carcinomas were analyzed: 71 adrenocortical carcinoma (ACC), 270 bladder urothelial carcinoma (BLCA), 228 breast invasive carcinoma (BRCA), 101 cervical squamous cell carcinoma (CESC), 196 head and neck squamous cell carcinoma (HNSC), 167 liver hepatocellular carcinoma (LIHC), 324 lung adenocarcinoma (LUAD), 118 lung squamous cell carcinoma (LUSC), 58 kidney chromophobe (KICH), 274 kidney renal clear cell carcinoma (KIRC), 149 kidney renal papillary cell carcinoma (KIRP), 46 pancreatic adenocarcinoma (PAAD), 349 prostate adenocarcinoma (PRAD), 181 stomach adenocarcinoma (STAD), 163 thyroid carcinoma (THCA), 112 uterine corpus endometrial carcinoma (UCEC). None of the patients were subjected to neoadjuvant therapies (neither chemotherapy or radiotherapy or immunotherapy) before tumor resection. A complete list of samples is given in Table S1. The effect mutations were predicted using cBioportal (Table S3) [42].

### 4.8. Pan-Cancer Characterization of Genomic Instability and Intratumor Heterogeneity

Genomic instability and ITH were determined using all the somatic point mutations and INDELs downloaded from the Broad Institute MAF dashboard. Genomic instability was calculated as the absolute number of mutations and INDEL observed in each tumor sample. The ITH defined as the genetic heterogeneity was measured considering the same somatic mutations and using the mutant-allele tumor heterogeneity (MATH) approach [24] (see Supplementary Methods for details). Briefly, for each individual tumor we: (1) obtained the mutant-allele fraction (MAF) values of the somatic mutations (the fraction of DNA that shows the mutated allele at a locus), (2) calculated the center (median) and the width of the distribution (median absolute deviation, MAD); (3) multiplied the median by a factor of 1.4826, so that the expected MAD of a normally distributed variable is equal to its standard deviation; (4) calculated the MATH value as the percentage ratio of the MAD to the median distribution of MAFs among the tumor’s genomic *loci* (MATH = 100 × MAD/median). Correlation between genomic instability and ITH was determined using Pearson method as implemented in cor.test function of R package [43].

### 4.9. Pan-Cancer Discovery of Driver-Gene Mutations of ITH

To identify driver-gene mutations, a binary matrix was produced representing the presence/absence of mutations for each gene on each tumor sample, eliminating the bias introduced by hypermutated genes. First, mutated genes were classified according to cancer specific pathways previously defined: epigenetic modifiers, transcription factors/regulators, genome integrity, RTK signaling, cell cycle, MAPK signaling, PI(3)K signaling, TGF-β signaling, Wnt/β-catenin signaling, proteolysis, splicing, HIPPO signaling, metabolism, NFE2L, protein phosphatase, ribosome, TOR [25]. By doing this, we reduced noise from passenger mutations and discover which group of genes is the major contributor of ITH in a wide range of carcinomas. Then, we applied a linear model per cancer type, extracting: explained variance, estimated coefficients, Benjamin-Hochberg adjusted *p*-values for the fitted model and for each estimated coefficient (Table S2). Second, to identify specific gene driver-events we used generalized linear models previously applied to infer association of genetic alterations with other variables [26] (see Supplementary Methods for details). Briefly, ITH for each individual cancer type and all cancers was modelled by Lasso regression as implemented in glmnet R package [44]. Significance of the explained variance by each model was determined for values greater than zero by a margin of more than one standard deviation. Finally, the fitted models were evaluated by comparing the observed and predicted ITH levels based on the tumor mutation profiles and assessing the Pearson correlation.

### 4.10. Whole-Exome Sequencing from Human Cancer Cell Lines

The genomic DNA from cells was prepared using the QIAamp DNA Mini Kit (Qiagen, Hilden, Germany) following the manufacturer’s instructions and the quality and quantity of purified DNA was assessed by NanoDrop™ 2000 (Thermo Fisher Scientific) and gel electrophoresis. Genomic DNA was extracted from control, DNMT3A and SETD2 KOs carcinoma cell lines following 1, 3 and 6 months in culture and then used for WES. Whole-exome capture libraries were constructed using 100 ng of DNA from Caki-2 cells (controls and KOs) sequenced as paired-end 151-bp sequence tags with coverage of 30×. Samples were barcoded and prepared for sequencing by GATC Biotech AG (www.gatc-biotech.com) using Illumina protocols. Integrity and quantity of the starting material was determined by appropriate methods (e.g., volume measurement, gel electrophoresis and fluorimeter measurements). Library preparation incorporated adaptor sequences and indexing compatible for Illumina sequencing technology, using proprietary methods of GATC Biotech. Enrichment was performed using Agilents SureSelectXT Human All Exon V6 technology. The quality of the final library was assessed by determination of size distribution and by quantification, following GATC Biotech protocols. Sequencing was carried out on the Illumina HiSeq platform. Delivered raw data is the result of a primary analysis using Illumina CASAVA software (http://cancan.cshl.edu/labmembers/gordon/fastq_illumina_filter/).

### 4.11. Variant Calling from Whole-Exome Sequencing

Whole-exome sequence data processing and analysis were performed by RubioSeq software (http://rubioseq.bioinfo.cnio.es/) using default parameters for somatic variation analysis [45]. Briefly, sequencing data were first checked by FastQC for quality control checks on raw sequence data and then aligned to the human reference genome (GRCh37/hg19) using Burrows-Wheeler alignment (BWA) [46]. Reads unmapped by BWA were realigned using BFAST [47]. Sequenced samples presented 71% of bases in the targeted exome above 30× coverage (see Supplementary Methods for details and Table S7). For variant calling we used GATK Unified Genotyper v2 [48] applying the “Discovery” genotyping mode and default parameters for filtering. The GATK QUAL field was employed for ranking selected somatic variants. Mutations were filtered to ensure that each variant had at least 5 reads supporting the mutant allele and coverage of ≥30. Single-nucleotide variants reported in dbSNP150 were filtered out from VCF output files, unless they were also present in COSMICv85 [49]. Only single nucleotide variants were used for downstream analyses. The filtered variants were annotated with SnpEff (VEP) [50]. Finally, to remove the germinal variants (i.e., present in the original cell population) we filtered out variants present in the earliest replicate (1 month) from each experiment (individual knockouts or control) and with MAF equal to 1.

### 4.12. Assessing ITH and Subclones Number from Whole-Exome Sequencing

The ITH from control and knockout cell lines was determined using the mutant-allele tumor heterogeneity (MATH) approach [24]. A Bayesian clustering approach was also used to infer clonal population structures present in control and knockout cell lines as implemented in Pyclone [30] (see Supplementary Methods for details). Pyclone analysis was performed jointly on all samples using variants supported at least by 50 reads and with copy number information estimated by RubioSeq and processed using CopyWriteR Bioconductor package [51].

### 4.13. Statistical Analysis and Graphical Representation

Figures were produced using ggplot R package [52] and default packages from R environment [43] and also Graph Pad Prism5 Software (https://www.graphpad.com/scientific-software/prism/). The statistical significance of differences between groups was evaluated using unpaired Student’s *t*-test and Mann-Whitney-Wilcoxon test (* *p* < 0.05; ** *p* < 0.01; *** *p* < 0.001; **** *p* < 0.0001). Results are depicted either as mean ± standard deviation (SD) or median ± SD, of minimum 3 independent replicates. Survival was analyzed by Kaplan-Meier curve comparison using a log-rank test and with a multivariate Cox proportional hazards analysis as implemented in the survival R package [53]. Statistical significance was determined using *p*-value < 0.05 as cut-off.

## 5. Conclusions

Our pan-cancer analyses revealed that mutations in epigenetic modifiers, namely *SETD2* and *DNMT3A,* are major determinants of ITH. These genes are recurrently mutated in several cancer types. For instance, *SETD2* mutations are found in 10% of KIRC [16], 9% of non-small cell lung carcinomas [54], 15% of pediatric high-grade gliomas and 8% of adult high-grade gliomas [55], whereas mutations in *DNMT3A* are observed in over 20% acute monocytic leukemias [15]. These numbers illustrate the broad significance of our findings, which provide an unprecedented pan-cancer portrait of the major determinants of ITH. Our experimental validation of the role of specific epigenetic modifier genes in driving ITH reveals novel biomarkers and/or therapeutic targets that may contribute to more effective cancer prognoses and treatment.

## Data Availability

WES sequencing data that correspond to human cell lines are available in the Sequence Read. Archive (SRA) with the following accession code: SRP153138.

## Supplementary Materials

The following are available online at https://www.mdpi.com/2072-6694/11/3/391/s1, Method S1: Pan-Cancer Data Sets, Method S2: Intratumor heterogeneity score using mutant-allele tumor heterogeneity (MATH) score, Method S3: Identification of deregulated cancer pathways associated with ITH, Method S4: Pan-cancer discovery of driver-gene mutations of ITH, Method S5: Whole-exome sequencing and variant calling for human cancer cell lines, Method S6: Clonality analyses. This file contains the description of the computational methods used in this study, Table S1: Clinical data and genomic features (genomic instability and MATH values) for samples from 16 different TCGA cancer types, Table S2: Linear models associating mutations in functional groups and ITH for each cancer type, Table S3: Predicted effect of DNMT1, DNMT3A and SETD2 mutations in KIRC samples from cBioportal, Table S4: ITH values (MATH) estimated for control, DNTM3A and SETD2 KO cell lines, Table S5: Mutations generated after SETD2 and DNMT3A knockouts (not present in controls), including the mutations with GO terms associated with mitochondria, Table S6: Material and Methods Table (e.g., plasmids, gRNAs, antibodies, primers and other products), Table S7: Read length, total reads and mapped reads for each condition.

## References

[1] Burrell R.A., McGranahan N., Bartek J., Swanton C. (2013). The causes and consequences of genetic heterogeneity in cancer evolution. Nature.

[2] Gerlinger M., McGranahan N., Dewhurst S.M., Burrell R.A., Tomlinson I., Swanton C. (2014). Cancer: Evolution Within a Lifetime. Annu. Rev. Genet..

[3] McGranahan N., Swanton C. (2017). Clonal Heterogeneity and Tumor Evolution: Past, Present, and the Future. Cell.

[4] Andor N., Graham T.A., Jansen M., Xia L.C., Aktipis C.A., Petritsch C., Ji H.P., Maley C.C. (2016). Pan-cancer analysis of the extent and consequences of intratumor heterogeneity. Nat. Med..

[5] Waclaw B., Bozic I., Pittman M.E., Hruban R.H., Vogelstein B., Nowak M.A. (2015). A spatial model predicts that dispersal and cell turnover limit intratumour heterogeneity. Nature.

[6] Cleary A.S., Leonard T.L., Gestl S.A., Gunther E.J. (2014). Tumour cell heterogeneity maintained by cooperating subclones in Wnt-driven mammary cancers. Nature.

[7] Turner K.M., Deshpande V., Beyter D., Koga T., Rusert J., Lee C., Li B., Arden K., Ren B., Nathanson D.A. (2017). Extrachromosomal oncogene amplification drives tumour evolution and genetic heterogeneity. Nature.

[8] Laughney A.M., Elizalde S., Genovese G., Bakhoum S.F. (2015). Dynamics of Tumor Heterogeneity Derived from Clonal Karyotypic Evolution. Cell Rep..

[9] Sotillo R., Schvartzman J.M., Socci N.D., Benezra R. (2010). Mad2-induced chromosome instability leads to lung tumour relapse after oncogene withdrawal. Nature.

[10] Hanahan D., Weinberg R.A. (2011). Hallmarks of cancer: The next generation. Cell.

[11] Berdasco M., Esteller M. (2010). Aberrant Epigenetic Landscape in Cancer: How Cellular Identity Goes Awry. Dev. Cell.

[12] Kaufman C.K., Mosimann C., Fan Z.P., Yang S., Thomas A.J., Ablain J., Tan J.L., Fogley R.D., van Rooijen E., Hagedorn E.J. (2016). A zebrafish melanoma model reveals emergence of neural crest identity during melanoma initiation. Science.

[13] Plass C., Pfister S.M., Lindroth A.M., Bogatyrova O., Claus R., Lichter P. (2013). Mutations in regulators of the epigenome and their connections to global chromatin patterns in cancer. Nat. Rev. Genet..

[14] Shen H., Laird P.W. (2013). Interplay between the cancer genome and epigenome. Cell.

[15] Yan X.J., Xu J., Gu Z.H., Pan C.M., Lu G., Shen Y., Shi J.Y., Zhu Y.M., Tang L., Zhang X.W. (2011). Exome sequencing identifies somatic mutations of DNA methyltransferase gene DNMT3A in acute monocytic leukemia. Nat. Genet..

[16] Creighton C., Morgan M., Gunaratne P., Wheeler D., Gibbs R., Robertson R., Chu A., Beroukhim R., Cibulskis K., Signoretti S. (2013). Comprehensive molecular characterization of clear cell renal cell carcinoma. Nature.

[17] Carvalho S., Vítor A.C., Sridhara S.C., Martins F.B., Raposo A.C., Desterro J.M., Ferreira J., de Almeida S.F. (2014). SETD2 is required for DNA double-strand break repair and activation of the p53-mediated checkpoint. Elife.

[18] Carvalho S., Raposo A.C., Martins F.B., Grosso A.R., Sridhara S.C., Rino J., Carmo-Fonseca M., de Almeida S.F. (2013). Histone methyltransferase SETD2 coordinates FACT recruitment with nucleosome dynamics during transcription. Nucleic Acids Res..

[19] Grosso A.R., Leite A.P., Carvalho S., Matos M.R., Martins F.B., Vítor A.C., Desterro J.M., Carmo-Fonseca M., de Almeida S.F. (2015). Pervasive transcription read-through promotes aberrant expression of oncogenes and RNA chimeras in renal carcinoma. Elife.

[20] Dhayalan A., Rajavelu A., Rathert P., Tamas R., Jurkowska R.Z., Ragozin S., Jeltsch A. (2010). The Dnmt3a PWWP domain reads histone 3 lysine 36 trimethylation and guides DNA methylation. J. Biol. Chem..

[21] Timp W., Feinberg A.P. (2013). Cancer as a dysregulated epigenome allowing cellular growth advantage at the expense of the host. Nat. Rev. Cancer..

[22] Hao J.J., Lin D.C., Dinh H.Q., Mayakonda A., Jiang Y.Y., Chang C., Jiang Y., Lu C.C., Shi Z.Z., Xu X. (2016). Spatial intratumoral heterogeneity and temporal clonal evolution in esophageal squamous cell carcinoma. Nat. Genet..

[23] Mazor T., Pankov A., Johnson B.E., Hong C., Hamilton E.G., Bell R.J., Smirnov I.V., Reis G.F., Phillips J.J., Barnes M.J. (2015). DNA Methylation and Somatic Mutations Converge on the Cell Cycle and Define Similar Evolutionary Histories in Brain Tumors. Cancer Cell.

[24] Mroz E.A., Rocco J.W. (2013). MATH, a novel measure of intratumor genetic heterogeneity, is high in poor-outcome classes of head and neck squamous cell carcinoma. Oral Oncol..

[25] Kandoth C., McLellan M.D., Vandin F., Ye K., Niu B., Lu C., Xie M., Zhang Q., McMichael J.F., Wyczalkowski M.A. (2013). Mutational landscape and significance across 12 major cancer types. Nature.

[26] Gerstung M., Pellagatti A., Malcovati L., Giagounidis A., Della Porta M.G., Jädersten M., Dolatshad H., Verma A., Cross N.C., Vyas P. (2015). Combining gene mutation with gene expression data improves outcome prediction in myelodysplastic syndromes. Nat. Commun..

[27] Yang L., Fang J., Chen J. (2017). Tumor cell senescence response produces aggressive variants. Cell Death Discov..

[28] Coppé J.-P., Desprez P.-Y., Krtolica A., Campisi J. (2010). The Senescence-Associated Secretory Phenotype: The Dark Side of Tumor Suppression. Annu. Rev. Pathol. Mech. Dis..

[29] Castro-Vega L.J., Jouravleva K., Ortiz-Montero P., Liu W.Y., Galeano J.L., Romero M., Popova T., Bacchetti S., Vernot J.P., Londoño-Vallejo A. (2015). The senescent microenvironment promotes the emergence of heterogeneous cancer stem-like cells. Carcinogenesis.

[30] Roth A., Khattra J., Yap D., Wan A., Laks E., Biele J., Ha G., Aparicio S., Bouchard-Côté A., Shah S.P. (2014). PyClone: Statistical inference of clonal population structure in cancer. Nat. Methods.

[31] Porporato P.E., Filigheddu N., Pedro J.M.B.-S., Kroemer G., Galluzzi L. (2017). Mitochondrial metabolism and cancer. Cell Res..

[32] Tan A.S., Baty J.W., Berridge M.V. (2014). The role of mitochondrial electron transport in tumorigenesis and metastasis. Biochim. Biophys. Acta.

[33] Alam M.M., Lal S., FitzGerald K.E., Zhang L. (2016). A holistic view of cancer bioenergetics: Mitochondrial function and respiration play fundamental roles in the development and progression of diverse tumors. Clin. Transl. Med..

[34] Cherry C., Thompson B., Saptarshi N., Wu J., Hoh J. (2016). A “Mitochondria” Odyssey. Trends Mol. Med..

[35] Raynaud F., Mina M., Tavernari D., Ciriello G. (2018). Pan-cancer inference of intra-tumor heterogeneity reveals associations with different forms of genomic instability. PLoS Genet..

[36] De S., Michor F. (2011). DNA secondary structures and epigenetic determinants of cancer genome evolution. Nat. Struct. Mol. Biol..

[37] Schuster-Böckler B., Lehner B. (2012). Chromatin organization is a major influence on regional mutation rates in human cancer cells. Nature.

[38] Pfister S.X., Ahrabi S., Zalmas L.P., Sarkar S., Aymard F., Bachrati C.Z., Helleday T., Legube G., La Thangue N.B., Porter A.C. (2014). SETD2-Dependent Histone H3K36 Trimethylation Is Required for Homologous Recombination Repair and Genome Stability. Cell Rep..

[39] Aymard F., Bugler B., Schmidt C.K., Guillou E., Caron P., Briois S., Iacovoni J.S., Daburon V., Miller K.M., Jackson S.P. (2014). Transcriptionally active chromatin recruits homologous recombination at DNA double-strand breaks. Nat. Struct. Mol. Biol..

[40] Gerlinger M., Rowan A.J., Horswell S., Larkin J., Endesfelder D., Gronroos E., Martinez P., Matthews N., Stewart A., Tarpey P. (2012). Intratumor Heterogeneity and Branched Evolution Revealed by Multiregion Sequencing. N. Engl. J. Med..

[41] Kanu N., Grönroos E., Martinez P., Burrell R.A., Goh X.Y., Bartkova J., Maya-Mendoza A., Mistrík M., Rowan A.J., Patel H. (2015). SETD2 loss-of-function promotes renal cancer branched evolution through replication stress and impaired DNA repair. Oncogene.

[42] Cerami E., Gao J., Dogrusoz U., Gross B.E., Sumer S.O., Aksoy B.A., Jacobsen A., Byrne C.J., Heuer M.L., Larsson E. (2012). The cBio Cancer Genomics Portal: An open platform for exploring multidimensional cancer genomics data. Cancer Discov..

[43] R Core Team (2018). R: A Language and Environment for Statistical Computing.

[44] Friedman J., Hastie T., Tibshirani R. (2010). Regularization Paths for Generalized Linear Models via Coordinate Descent. J. Stat. Softw..

[45] Rubio-Camarillo M., Gómez-López G., Fernández J.M., Valencia A., Pisano D.G. (2013). RUbioSeq: A suite of parallelized pipelines to automate exome variation and bisulfite-seq analyses. Bioinformatics.

[46] Li H., Durbin R. (2009). Fast and accurate short read alignment with Burrows-Wheeler transform. Bioinformatics.

[47] Homer N., Merriman B., Nelson S.F. (2009). BFAST: An alignment tool for large scale genome resequencing. PLoS ONE.

[48] DePristo M.A., Banks E., Poplin R., Garimella K.V., Maguire J.R., Hartl C., Philippakis A.A., Del Angel G., Rivas M.A., Hanna M. (2011). A framework for variation discovery and genotyping using next-generation DNA sequencing data. Nat. Genet..

[49] Forbes S.A., Beare D., Boutselakis H., Bamford S., Bindal N., Tate J., Cole C.G., Ward S., Dawson E., Ponting L. (2017). COSMIC: Somatic cancer genetics at high-resolution. Nucleic Acids Res..

[50] Cingolani P., Platts A., Wang L.L., Coon M., Nguyen T., Wang L., Land S.J., Lu X., Ruden D.M. (2012). A program for annotating and predicting the effects of single nucleotide polymorphisms, SnpEff: SNPs in the genome of Drosophila melanogaster strain w1118; iso-2; iso-3. Fly.

[51] Kuilman T., Velds A., Kemper K., Ranzani M., Bombardelli L., Hoogstraat M., Nevedomskaya E., Xu G., de Ruiter J., Lolkema M.P. (2015). CopywriteR: DNA copy number detection from off-target sequence data. Genome Biol..

[52] Wickham H. (2016). ggplot2: Elegant Graphics for Data Analysis. eBook.

[53] Therneau T., Grambsch P. (2002). Modeling Survival Data: Extending the Cox Model. Technometrics.

[54] Govindan R., Ding L., Griffith M., Subramanian J., Dees N.D., Kanchi K.L., Maher C.A., Fulton R., Fulton L., Wallis J. (2012). Genomic landscape of non-small cell lung cancer in smokers and never-smokers. Cell.

[55] Fontebasso A.M., Schwartzentruber J., Khuong-Quang D.A., Liu X.Y., Sturm D., Korshunov A., Jones D.T., Witt H., Kool M., Albrecht S. (2013). Mutations in SETD2 and genes affecting histone H3K36 methylation target hemispheric high-grade gliomas. Acta Neuropathol..

