# Supplementary material for: A Systematic Pan-Cancer Analysis of Genetic Heterogeneity Reveals Associations with Epigenetic Modifiers
Source: Cancers (Basel). 2019 Mar 20;11(3):391. doi: 10.3390/cancers11030391 (PMC6468518; doi:10.3390/cancers11030391)
Supplement: Supplementary file 1 [file cancers-11-00391-s001.zip › cancers-454401 - supplementary file 3.21/cancers-454401 - Supplementary - final check.pdf]

Supplementary Materials: A Systematic Pan-Cancer Analysis of Genetic Heterogeneity Reveals Associations with Epigenetic Modifiers

Mafalda Ramos de Matos, Ioana Posa, Filipa Sofia Carvalho, Vanessa Alexandra Morais, Ana Rita Grosso and Sérgio Fernandes de Almeida

Supplementary Methods

Method S1: Pan-Cancer Data Sets

The TCGA consortium performed exome sequencing on thousands of tumor samples and matched normal tissues, the latter being used as controls to distinguish somatic mutations from inherited variants. These controls were peripheral blood and, in some cases, adjacent solid normal tissue. Tumor-specific mutation data from whole-exome sequencing (WES) were downloaded from publicly available somatic variant calls in mutation annotation format (MAF) files from the TCGA. MAF files were downloaded from Broad Institute MAF dashboard (https://confluence.broadinstitute.org/display/GDAC/MAF+Dashboard), released (14 April 2017). Mutation data were available for 2807 patients with clinical data, corresponding to 16 different carcinomas: 71 adrenocortical carcinoma (ACC), 270 bladder urothelial carcinoma (BLCA), 228 breast invasive carcinoma (BRCA), 101 cervical squamous cell carcinoma (CESC), 196 head and neck squamous cell carcinoma (HNSC), 167 liver hepatocellular carcinoma (LIHC), 324 lung adenocarcinoma (LUAD), 118 lung squamous cell carcinoma (LUSC), 58 kidney chromophobe (KICH), 274 kidney renal clear cell carcinoma (KIRC), 149 kidney renal papillary cell carcinoma (KIRP), 46 pancreatic adenocarcinoma (PAAD), 349 prostate adenocarcinoma (PRAD), 181 stomach adenocarcinoma (STAD), 163 thyroid carcinoma (THCA), 112 uterine corpus endometrial carcinoma (UCEC). From clinical dataset we selected only the patients which were subjected to neoadjuvant therapies before tumor resection. A complete list of samples is given in Table S1. The effect of mutations was predicted using cBioportal (Table S3) [1]. Also, genomic instability and ITH were determined using all the somatic point mutations and INDELs downloaded from the Broad Institute MAF dashboard.

Method S2: Intratumor Heterogeneity Score Using Mutant-Allele Tumor Heterogeneity (MATH) Score

Intratumor heterogeneity (ITH) defined as the genetic heterogeneity was measured considering somatic mutations (point mutations and INDELs) and using the mutant-allele tumor heterogeneity (MATH) approach [2]. Genomic *loci* having tumor-specific somatic mutations were identified, based on tumor-normal matched paired DNA. Then, MAF was determined for each locus as the ratio of mutant reads to total reads, and tabulated all MAF values (as the fraction of DNA that shows the mutated allele at a *locus*). We restricted our analysis to *loci* having MAFs above 0.075 with no further restrictions in the analysis. For each tumor we then determined the median and the median absolute deviation (MAD) of its MAF values. The median is a robust measure of the center of the distribution of MAFs. The MAD is a strong measure of the width of the distribution that is much less sensitive to outliers than the standard deviation (SD), and is determined as follow, the absolute value of the difference of each MAF from the median MAF value is calculated, and the median of those absolute differences is taken. This median difference is then multiplied by a factor of 1.4826, so that the expected MAD of a normally distributed variable is equal to its SD. Finally, the MATH value for each tumor was calculated as the percentage ratio of the MAD to the median of the distribution of MAFs among the tumor's mutated genomic *loci*:

$$\text{MATH} = 100 \times \text{MAD/median}$$

Simply using the width of the distribution as a measure of genomic heterogeneity would not consider the overall lowering of MAF values by the "impurity" of normal DNA in the tumor sample. As previously described, dividing the MAD by the median provides a first-order correction for this "impurity," as a more "impure" sample is expected to have a lower median MAF value. Each tumor's MATH value was calculated from the median absolute deviation (MAD) and the median of its mutant-allele fractions at tumor-specific mutated *loci*. Importantly, using this method we need to have into account that high number of mutations/genomic instability does not imply high ITH (Figure S1).

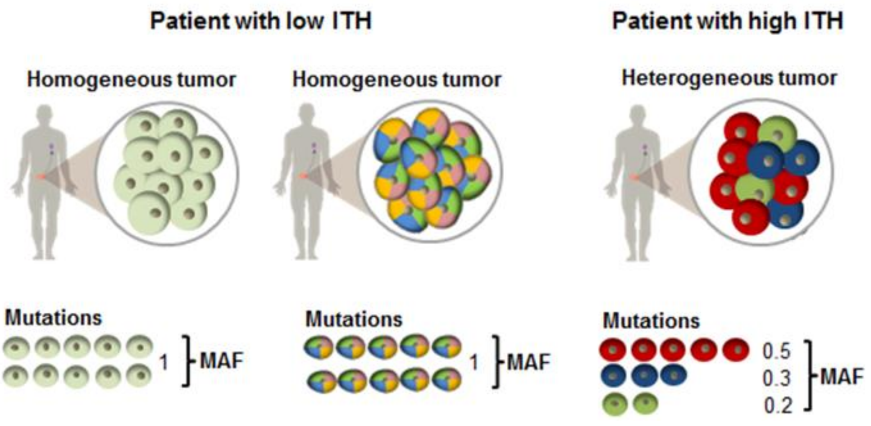

Figure S1: Example of homogeneous and heterogeneous tumors defined using the MATH score. In the first example of a homogeneous tumor all cells share the same mutation (light green represents the mutant allele) and the MAF is equal to 1. In the second situation, the tumor has four mutant alleles represented by the different colors. The mutations are equally shared by all cells from the tumor, MAF is 1 and the tumor is also considered homogeneous. Lastly, the case of a heterogeneous tumor, where different mutations are found with different frequencies. For instance, MAF is 0.5 for red, 0.3 for the blue and 0.2 for the green locus. According with the MATH score, the distribution of mutant-allele fractions from the heterogeneous tumor is wider (larger MAD) and has a lower median than for the homogeneous tumors. The ratio of the width to the center of the distribution (MATH = 100 × MAD/median) is thus larger for the heterogeneous tumor.

In Figure 1, violin plots show genomic instability (upper panel) and ITH score (bottom panel) within tumor types, across 16 cancer types. The width of the violins is proportional to the genomic instability and ITH, respectively and the black circle shows the median value observed for each tumor type (Figure 1A). Cancer types were ordered according to the extent of genomic instability (log10 transformed). Correlations of genomic instability with ITH scores were done using Pearson method as implemented in cor.test function of R package. For each tumor type regressions, scatterplots and linear regressions were evaluated. Regression coefficients "*r*" and *p*-values were written in the scatterplots for each individual cancer (Figure 1B).

Method S3: Identification of Deregulated Cancer Pathways Associated with ITH

In order to reduced noise from passenger mutations and discover which group of genes is the major contributor of ITH, we classified mutated genes according to cancer specific pathways previously defined: epigenetic modifiers, transcription factors/regulators, genome integrity, RTK signaling, cell cycle, MAPK signaling, PI(3)K signaling, TGF-β signaling, Wnt/β-catenin signaling, proteolysis, splicing, HIPPO signaling, metabolism, NFE2L, protein phosphatase, ribosome and TOR [3]. To identify driver-gene mutations a binary matrix was produced representing the presence/absence of mutations for each gene on each tumor sample, eliminating the bias introduced by hypermutated genes. We applied a linear model (https://www.rdocumentation.org/packages/stats/versions/3.5.3/topics/lm) per cancer type,

extracting: explained variance, estimated coefficients, Benjamin-Hochberg adjusted p-values for the fitted model and for each estimated coefficient (Figure 2C,D and Table S2).

We used the standard Cox proportional hazards model for individual cancer types, as implemented in the R package 'survival' (http://cran.r-project.org/web/packages/survival/). Significance analysis of hazard ratios (HRs) used the Wald test. Statistical significance was accepted at p-value < 0.05 in two-sided tests. Hazard ratios exceeding 1 indicate an overall detrimental effect across cancers, whereas those below 1 associate with better outcome (Figure 2B).

Method S4: Pan-Cancer Discovery of Driver-Gene Mutations of ITH

Mutations in specific genes associated with cancer and ITH score were modelled by generalized linear model via penalized maximum likelihood (Lasso penalized model) [4]. The approach was used to model the ITH levels for each cancer type independently (MATH values) according to the mutations in epigenetic modifier genes (binary matrix representing the presence/absence of mutations for each gene on each tumor sample) using generalized linear function as implemented glmnet R package [5]. Only significant fitted models were selected for downstream analyses. The variance explained by each fitted model was determined with the coefficient of determination R2, for which the statistical significance was determined for values greater than zero by a margin of more than one standard deviation. The significant Lasso penalized models identified for each cancer type were graphically represented using a composite heatmap (Figure 2E). First, Lasso-selected coefficients were colored according to the effect of each standardized covariate in the optimal model. The numbers on each tile denote the order in which variables are included in the model indicating their relative importance. The top bar plot indicates the frequency that a driver-gene mutation appears as significantly contributing for the ITH fitted model. The right bar plot shows the explained variance for each model. Finally, the fitted models were evaluated by comparing the observed and predicted ITH levels based on the tumor mutation profiles and assessing the Pearson correlation (Figure 2G).

Method S5: Whole-Exome Sequencing and Variant Calling For Human Cancer Cell Lines

Whole-exome capture libraries were constructed from 100 ng of DNA from Caki-2 control and knock-out cells, sequenced as paired-end 151-bp sequence tags (Table S7) and subjected to exonic hybrid capture using Agilents SureSelectXT Human All Exon V6 technology. Samples were barcoded and prepared for sequencing by GATC Biotech AG (www.gatc-biotech.com) using Illumina protocols. Samples were subjected of volume measurement, gel electrophoresis and fluorimeter measurements, in order to assure integrity and quantity of the starting material.

Sequence data processing and quality control whole-exome sequence data processing and analysis were performed were performed by RubioSeq software (http://rubioseq.bioinfo.cnio.es/, v3.8). Rubioseq pipeline assesses data quality, performs read alignment and variant calling using the state-of-art methodologies [6]. Briefly, Illumina reads were aligned to the reference human genome build (GRCh37/hg19) using an implementation of the Burrows-Wheeler Aligner (BWA) [7]. Reads unmapped by BWA were realigned using BFAST. Sequenced samples presented 71% of bases in the targeted exome above 30× coverage (Figure S2). For variant calling we used GATK Unified Genotyper v2 [8] applying the "Discovery" genotyping mode and default parameters for filtering. The GATK QUAL field was employed for ranking selected somatic variants. Mutations were filtered to ensure that each variant had at least 5 reads supporting the mutant allele and coverage of ≥30. Single-nucleotide variants reported in dbSNP150 were filtered out from VCF output files, unless they were also present in COSMIC (v85) [9]. The filtered variants were annotated with SnpEff (4.3m version) [10].

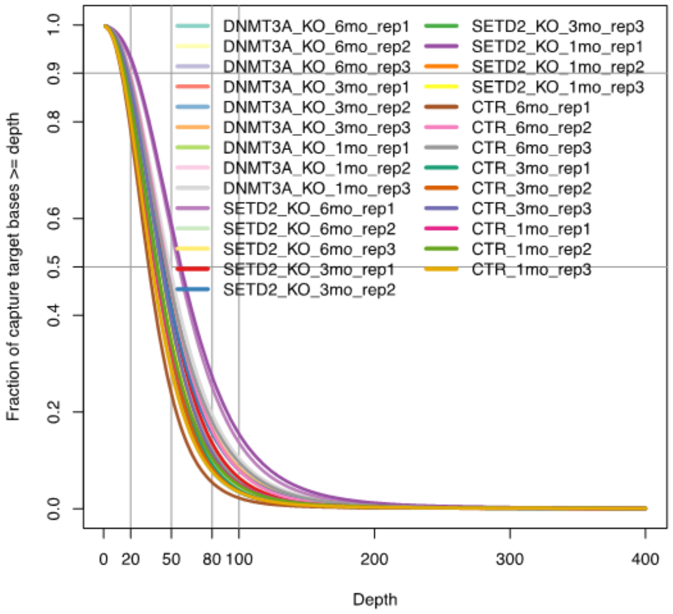

Figure S2. Sequencing coverage across control and knock-out samples.

Method S6: Clonality Analyses

Subclones were obtained by evaluating variant allele frequencies (VAFs) with Pyclone (v0.13.0), which deconvolves tumor sequences into subclones based on hierarchical Bayesian clustering model [11]. Pyclone analysis was performed jointly on all samples using variants supported at least by 50 reads and with copy number information estimated by CopyWriteR Bioconductor package [12]. Pyclone was run using the run_analysis_pipeline command with 10,000 iterations of the Markov Chain Monte Carlo chain.

Supplementary Tables 1–7: Please see supplementary files.

References

1. Cerami, E.; Gao, J.; Dogrusoz, U.; Gross, B.E.; Sumer, S.O.; Aksoy, B.A.; Jacobsen, A.; Byrne, C.J.; Heuer, M.L.; Larsson, E.; et al. The cBio Cancer Genomics Portal: An open platform for exploring multidimensional cancer genomics data. *Cancer Discov.* **2012**, *2*, 401–404, doi:10.1158/2159-8290.CD-12-0095.
2. Mroz, E.A.; Rocco, J.W. MATH, a novel measure of intratumor genetic heterogeneity, is high in poor-outcome classes of head and neck squamous cell carcinoma. *Oral Oncol.* **2013**, *49*, 211–215, doi:10.1016/j.oraloncology.2012.09.007.
3. Kandoth, C.; McLellan, M.D.; Vandin, F.; Ye, K.; Niu, B.; Lu, C.; Xie, M.; Zhang, Q.; McMichael, J.F.; Wyczalkowski, M.A.; et al. Mutational landscape and significance across 12 major cancer types. *Nature* 2013, *502*, 333–339, doi:10.1038/nature12634.
4. Gerstung, M.; Pellagatti, A.; Malcovati, L.; Giagounidis, A.; Della Porta, M.G.; Jädersten, M.; Dolatshad, H.; Verma, A.; Cross, N.C.; Vyas, P.; et al. Combining gene mutation with gene expression data improves outcome prediction in myelodysplastic syndromes. *Nat. Commun.* **2015**, *6*, 5901, doi:10.1038/ncomms6901.
5. Friedman, J.; Hastie, T.; Tibshirani, R. Regularization Paths for Generalized Linear Models via Coordinate Descent. *J. Stat. Softw.* **2010**, *33*, doi:10.18637/jss.v033.i01.
6. Rubio-Camarillo, M.; Gómez-López, G.; Fernández, J.M.; Valencia, A.; Pisano, D.G. RUbioSeq: A suite of parallelized pipelines to automate exome variation and bisulfite-seq analyses. *Bioinformatics* **2013**, *29*, 1687–1689, doi:10.1093/bioinformatics/btt203.
7. Li. H.; Durbin, R. Fast and accurate short read alignment with Burrows-Wheeler transform. *Bioinformatics* **2009**, *25*, 1754–1760, doi:10.1093/bioinformatics/btp324.
8. Depristo, M.A.; Banks, E.; Poplin, R.; Garimella, K.V.; Maguire, J.R.; Hartl, C.; Philippakis, A.A.; del Angel, G.; Rivas, M.A.; Hanna, M.; et al. A framework for variation discovery and genotyping using next-generation DNA sequencing data. *Nat. Genet.* **2011**, *43*, 491–498, doi:10.1038/ng.806

9. Forbes, S.A.; Beare, D.; Boutselakis, H.; Bamford, S.; Bindal, N.; Tate, J.; Cole, C.G.; Ward, S.; Dawson, E.; Ponting, L.; et al. COSMIC: Somatic cancer genetics at high-resolution. *Nucleic Acids Res.* **2017**, *45*, D777–D783, doi:10.1093/nar/gkw1121.
10. Cingolani, P.; Platts, A.; Wang, L.L.; Coon, M.; Nguyen, T.; Wang, L.; Land, S.J.; Lu, X.; Ruden, D.M. A program for annotating and predicting the effects of single nucleotide polymorphisms, SnpEff: SNPs in the genome of Drosophila melanogaster strain w1118; iso-2; iso-3. *Fly* **2012**, *6*, 80–92, doi:10.4161/fly.19695.
11. Roth, A.; Khattra, J.; Yap, D.; Wan, A.; Laks, E.; Biele, J.; Ha, G.; Aparicio, S.; Bouchard-Côté, A.; Shah, S.P. PyClone: Statistical inference of clonal population structure in cancer. *Nat. Methods* **2014**, *11*, 396–398, doi:10.1038/nmeth.2883.
12. Kuilman, T.; Velds, A.; Kemper, K.; Ranzani, M.; Bombardelli, L.; Hoogstraat, M.; Nevedomskaya, E.; Xu, G.; de Ruiter, J.; Lolkema, M.P.; et al. CopywriteR: DNA copy number detection from off-target sequence data. *Genome Biol.* **2015**, *16*, doi:10.1186/s13059-015-0617-1.

© 2019 by the authors. Licensee MDPI, Basel, Switzerland. This article is an open access article distributed under the terms and conditions of the Creative Commons Attribution (CC BY) license (http://creativecommons.org/licenses/by/4.0/).
